# Lint and Oakum in Gun-Shot and Other Suppurating Wounds

**Published:** 1862-11

**Authors:** 


### EDITORIAL DEPARTMENT.

### LINT AND OAKUM IN GUN-SHOT AND OTHER SUPPURATING WOUNDS.

The only object of lint or oakum applied to a suppurating wound, is to absorb the discharge; and when made of cotton or linen, and pressed upon the opening, is more likely to prevent the free escape of pus, than to absorb it. It is claimed by those having extensive opportunity of observation that oakum is much superior to lint for this purpose, and free from the objection of restraining or obstructing the discharge, while the cost is comparatively nothing. In cases where extensive suppuration is taking place, oakum, old worn linen, bran, saw dust, or other material may be convenient and necessary in large quantity, to absorb the pus and lessen the necessary care of attendants, the material being best for the purpose which will most readily absorb the discharge. The Surgeon-General recently issued a call for lint, and directions for its preparation, since which time the whole female and juvenile population have been engaged in scraping or separating the threads of worn cotton and linen, and packing it in boxes for the supply of the army. Little bundles of single threads, the size of a finger in length and thickness, have been made with great outlay of vital force and most commendable exhibition of patience.

In our recent visit to the hospitals in the District of Columbia, we made diligent effort to discover if there was really reason for scraping and picking in shreds, pieces of worn linen, and nowhere in a single instance did we observe any use of the scraped lint or bundles of threads. Oakum was applied in very few instances, but in quite small amount, and apparently without any definite object.

The small, square pieces of old cloth, such as are being subjected to the picking process, are in constant use, and are very indispensable in the care of the thousands of simple gun-shot wounds; but the boxes of lint as it is prepared, look more like a recently imported box of millinery goods, than anything for the use of the army.

It appears to us that lint as called for by the Surgeon-General, must have had reference mainly to the worn cotton or linen, cut in pieces suitable for dressing the gun-shot wounds, and that no part be thrown away, it was requested that the threads, &c., be saved, and sent also. However this may be, we hold to the private opinion that the unscraped cloth is vastly more useful than what is popularly regarded as lint. Lint, surgically speaking, is loose, soft cloth, used for absorbing the discharge in suppurating wounds. Old worn cloth is lint, or may be used as lint in place of that manufactured for surgical purposes, at a cost of about $1.50 per pound. This is not so much a matter of interest to physicians as to the public generally, and from the repeated enquiries made of us by ladies and others, we have no doubt that a little explanation would be acceptable. The first military rule we believe is, to "obey orders," whatever that may be; this we sufficiently approve, and have only hoped, if not ourselves mistaken, to aid in properly understanding them.

